# Supplementary material for: Aberrant Dynamic Functional Connectivity of Default Mode Network in Schizophrenia and Links to Symptom Severity
Source: Front Neural Circuits. 2021 Mar 18;15:649417. doi: 10.3389/fncir.2021.649417 (PMC8013735; doi:10.3389/fncir.2021.649417)
Supplement: Supplementary file 1 [file Data_Sheet_1.PDF]

Supplementary Information

Aberrant Dynamic Functional Connectivity of Default Mode Network in Schizophrenia and Links to Symptom Severity

Mohammad S.E. Sendi[1-3*], Elaheh Zendehrouh[4*], Charles A. Ellis[1,3], Zhijia Liang[3], Zening Fu[3], Daniel H. Mathalon[5,6], Judith M. Ford[5,6], Adrian Preda[7], Theo G. M. van Erp[7], Robyn. L Miller[3,4], Godfrey D. Pearlson[8], Jessica A. Turner[3,9†], Vince D. Calhoun[1-4,8,9†]

[1] *Wallace H. Coulter Department of Biomedical Engineering at Georgia Institute of Technology and Emory University, Atlanta, GA, United States.*

[2] *Department of Electrical and Computer Engineering at Georgia Institute of Technology, Atlanta, GA, United States.*

[3] *Tri-institutional Center for Translational Research in Neuroimaging and Data Science, Georgia State University, Georgia Institute of Technology, Emory University, Atlanta, GA, United States.*

[4] *Department of Computer Science at Georgia State University, Atlanta, GA, United States.*

[5] *Department of Psychiatry and Weill Institute for Neurosciences, University of California, San Francisco, CA, United States.*

[6] *Mental Health Service, Veterans Affairs San Francisco Healthcare System, San Francisco, CA, United States.*

[7] *Department of Psychiatry and Human Behavior, University of California, Irvine, Irvine, CA, United States.*

[8] *Department of Psychiatry, Yale University, School of Medicine, New Haven, CT, United States.*

[9] *Department of Psychology, Neuroscience Institute, Georgia State University, Atlanta, GA, United States.*

E-mail: vcalhoun@gsu.edu (Corresponding author: Vince D. Calhoun)
*Equal contribution as first authors
†Equal contribution as senior authors

Classification and feature selection results for each state

For the COBRE dataset, the area under the receiver operating characteristic curve (AUC) was 0.72±0.02. 0.66±0.01, 0.76±0.02, 0.71±0.02, and 0.66±0.03 in state 1, state 2, state 3, state 4, and state 5, respectively (Figure 4). The feature learning results showed that C_3 (the connectivity between PCu1 and ACC1 or PCu1/ACC1), C_{10} (PCu2/PCC1), C_{19} (ACC2/PCC1) were the most important features in the classification between SZ and HC in state 1 (corrected $p<0.05$). For state 2, the most important features were C_1 (PCu1/PCu2), C_5 (PCu1/PCC1), C_7 (PCu2/PCu3), C_8 (PCu2/ACC1), C_{10} (PCu2/PCC1), C_{12} (PCu3/ACC1), C_{15} (PCu3/PCC2), C_{16} (ACC1/ACC2), and C_{19} (ACC2/PCC1) (corrected $p<0.05$). In state 3, C_7 (PCu2/PCu3), C_9 (PCu2/ACC2), C_{12} (PCu3/ACC1), and C_{14} (PCu3/PCC1) were the most important features in SZ vs. HC classification (corrected $p<0.05$). C_1 (PCu1/PCu2), C_9 (PCu2/ACC2), C_{10} (PCu2/PCC1), C_{11} (PCu2/PCC2), and C_{19} (ACC2/PCC1) were the most important features in the state 4 classification (corrected $p<0.05$). In state5, C_8 (PCu2/ACC1), C_{11} (PCu2/PCC2), C_{15} (PCu3/PCC2), C_{17} (ACC1/PCC1), and C_{20} (ACC2/PCC2) most contributed to the classification between SZ and HC (corrected $p<0.05$).

A similar feature selection method on connectivity classification between SZ subjects and HC was conducted for the FBIRN dataset, as shown in Figure 5. The classification AUC between SZ and HC was 0.72±0.01, 0.68±0.01, 0.67±0.02, 0.69±0.01, and 0.68±0.02 in state 1, state 2, state 3, state 4, and state 5, respectively. C_1 (PCu1/PCu2), C_6 (PCu1/PCC2), C_{10} (PCu2/PCC1), C_{14} (PCu3/PCC1), C_{16} (ACC1/ACC2), and C_{20} (ACC2/PCC2) were the most important features in the discrimination between HC and SZ in state 1. In state 2, C_{11} (PCu2/PCC2), C_{15} (PCu3/PCC2), C_{16} (ACC1/ACC2), C_{18} (ACC1/PCC2), and C_{19} (ACC2/PCC1) were the most important features in the classification between HC and SZ subjects. In state3, C_5 (PCu1/PCC1), C_9 (PCu2/ACC2), C_{12} (PCu3/ACC1), C_{14} (PCu3/PCC1), C_{16} (ACC1/ACC2) were the most important features (corrected

$p<0.05$). In state 4, C_5 (PCu1/PCC1), C_8 (PCu2/ACC1), C_9 (PCu2/ACC2), C_{14} (PCu3/PCC1), C_{16} (ACC1/ACC2), C_{19} (ACC2/PCC1) were the most important features in the classification between HC and SZ (corrected $p<0.05$). In state 5, the most important features were C_{10} (PCu2/PCC1), C13 (PCu3/ACC2), C_{14} (PCu3/PCC1), C_{16} (ACC1/ACC2), C_{18} (ACC1/PCC2), C_{19} (ACC2/PCC1), and C_{20} (ACC2/PCC2).

Supplemental Table S1

Demographic and clinical details of FBIRN subjects for each site.

		SZ	*HC*	*P-value*
FBIRN Site 1	**Number**	21	28	NA
	**Age**	30.04±8.60	34.78±9.40	0.1
	**Gender(M/F)**	17/4	21/7	0.99
	**PANSS (positive)**	15.72±5.57	NA	NA
	**PANSS(negative)**	14.11±3.14	NA	NA
FBIRN Site 2	**Number**	12	10	NA
	**Age**	44.91±11.34	38.10±9.39	0.14
	**Gender(M/F)**	12/0	7/3	0.62
	**PANSS (positive)**	16.90±6.70	NA	NA
	**PANSS negative)**	17.20±7.39	NA	NA
FBIRN Site 3	**Number**	24	27	NA
	**Age**	44.41±11.90	42.48±12.56	0.57
	**Gender(M/F)**	19/5	21/6	0.99
	**PANSS (positive)**	16.95±4.43	NA	NA
	**PANSS negative)**	16.87±5.91	NA	NA
FBIRN Site 4	**Number**	26	26	NA
	**Age**	36.88±12.82	35.23±10.56	0.61
	**Gender(M/F)**	21/5	20/6	0.99
	**PANSS (positive)**	14.29±3.77	NA	NA
	**PANSS (negative)**	13.41±4.64	NA	NA
FBIRN Site 5	**Number**	14	15	NA
	**Age**	36.64±10.27	37.53±9.76	0.81
	**Gender(M/F)**	9/5	10/5	0.99
	**PANSS (positive)**	13.14±4.46	NA	NA
	**PANSS (negative)**	15±6.28	NA	NA
FBIRN Site 6	**Number**	29	27	NA
	**Age**	36.27±11.08	34.51±10.85	0.87
	**Gender(M/F)**	17/12	16/9	0.99
	**PANSS (positive)**	14.34±4.67	NA	NA
	**PANSS (negative)**	11.93±4.06	NA	NA
FBIRN Site 7	**Number**	25	27	NA
	**Age**	39.56±11.80	37.56±10.75	0.52
	**Gender(M/F)**	20/5	20/7	0.99
	**PANSS (positive)**	16.12±5.41	NA	NA
	**PANSS (negative)**	14.04±6.82	NA	NA

Note: SZ: Schizophrenia; HC: healthy control; PANSS: Positive and Negative Syndrome Scale, M: Male, F: Female, NA: not applicable; all p values have been calculated using two-sample t-test.
